# The Study of Serum Vitamin D and Insulin Resistance in Chinese Populations with Normal Glucose Tolerance

**DOI:** 10.1155/2014/870235

**Published:** 2014-09-01

**Authors:** Lin Ding, Congcong Wang, Heliang Ma, Yuling Tian, Yong Lu, Shuguang Pang

**Affiliations:** ^1^Department of Endocrinology, Jinan Central Hospital Affiliated to Shandong University, No. 105 Jie Fang Road, Jinan, Shandong 250013, China; ^2^Department of Endocrinology, Shengze Branch of Jiangsu Provincial Hospital, Wujiang 215228, China

## Abstract

*Objectives*. The aim of this study was to investigate the relationship between serum vitamin D and insulin resistance in Chinese subjects without diabetes mellitus. *Methods*. Serum 25(OH)D was measured in 897 individuals with normal glucose tolerance (NGT). Oral glucose tolerance tests (OGTTs) were conducted to exclude cases with diabetes, impaired fasting glucose (IFG), and impaired glucose tolerance (IGT). Metabolic parameters were measured and compared between the highest and lowest 25(OH)D quartiles. The relationship between serum 25(OH)D and homeostatic model assessment-insulin resistance (HOMA-IR) was analyzed. *Results*. Indexes, such as HOMA-IR, FINS, and SBP, were negatively correlated with serum 25(OH)D concentrations. Compared with the lowest quartile, individuals in the highest group had decreased Lg (HOMA-IR), Lg (FINS), and SBP. Pearson correlation analyses showed that serum 25(OH)D was negatively associated with age, BMI, Lg (HOMA-IR), and Lg (FINS). Multivariate linear regression analysis confirmed the negative correlation of Lg (HOMA-IR) and 25(OH)D. *Conclusions*. This study showed that serum 25(OH)D could be regarded as an independent predictor of insulin resistance for subjects without diabetes mellitus in China. Adequate vitamin D supplementation may improve multiple metabolic disturbances.

## 1. Introduction

The importance of vitamin D in the maintenance of calcium homeostasis and bone health is well known. In recent years, there is a growing interest in the nonclassical effects of vitamin D, which is based on findings showing the presence of vitamin D receptors (VDRs) in tissues other than bone, gut, and kidneys. The previous studies had shown that vitamin D status was correlated with a plethora of adverse health conditions such as endocrine [1], cardiovascular diseases [2], cancer [3], and autoimmune diseases [4]. Patients with type 2 diabetes had lower serum 25(OH)D compared to control subjects without diabetes [5]. The 25(OH)D concentrations were lower in subjects at risk of diabetes than those without the risk. Moreover, vitamin D supplementation showed beneficial effects on glucose homeostasis and insulin resistance both in patients with type 2 diabetes and in adults at risk of type 2 diabetes [6, 7].

The study on the relationship between serum 25(OH)D and insulin resistance in subjects with NGT is insufficient currently. First of all, the previous studies mainly focus on the Western populations as well as established diabetic subjects, while those conducted among Asian subjects free of diabetes are much less [8, 9]. Besides, existing results from observational and experimental studies are not entirely consistent [10–14]. Scragg et al. [10] had shown racial/ethnic disparities in the associations of 25(OH)D with insulin resistance in USA adults. Our study aimed to investigate the relationship between serum vitamin D and insulin resistance in Chinese populations with NGT.

## 2. Subjects and Methods

### 2.1. Study Participants

Study participants were individuals who were referred to our hospital for physical examinations from August to November in 2011. A total of 897 subjects aged 27–68 years, including 485 males and 412 females, with normal glucose tolerance were enrolled. Subjects were excluded in the following situations: diabetes, IFG and IGT, digestive system diseases leading to malabsorption, liver or kidney diseases, osteoporosis, and other abnormalities in bone metabolism. All participants denied a history of vitamin D and calcium intake. The 2003 ADA diagnostic criteria for diabetes and for lesser degrees of impaired glucose regulation (IFG/IGT) were adopted.

Protocols were approved by review boards of the Jinan Central Hospital. Written informed consent and investigations were conducted according to the principles in the Declaration of Helsinki.

### 2.2. Methods

Height was measured to the nearest cm using a rigid stadiometer and weight was measured in underwear to the nearest 0.1 kg using a calibrated balance scale. Body mass index (BMI) was calculated as weight (kg) divided by squared height (m^2^). Blood pressure was measured according to standard methods by special trainees. After an 8–12-hour overnight fast, a 75-g OGTT was administered, subjects with IFG, IGT, and diabetes were excluded, and those with NGT were enrolled according to 2003 American Diabetes Association criteria. 0-hour and 2-hour OGTT plasma glucose samples were measured with a hexokinase reagent kit (A-gent glucose test, Abbott Laboratories). Glucose assays were performed in duplicate; intra-assay CV was <3%. Fasting plasma insulin (FINS) was measured with an insulin assay specific to insulin (Linco Research); the assay CV was <6.8%. Indicators, such as triacylglycerol (TG) and total cholesterol (TCH), were measured by the enzymatic method. Measurement of HDL-cholesterol (HDLC) and LDL-cholesterol (LDLC) was conducted by the direct method. Serum 25(OH)D concentrations were measured by enzyme-linked immunosorbent assays (Immunodiagnostic Systems Limited, Boldon, UK) with intra-assay and interassay CVs of <6%. HOMA-IR is measured as fasting plasma insulin (mIU/L) × fasting plasma glucose (mmol/L)/22.5.

### 2.3. Groups

Serum 25(OH)D values were categorized into quartiles. Metabolic indexes were compared between 225 individuals (180 males and 45 females) in the highest quartile [serum 25(OH)D > 39.33 nmol/L] and 224 subjects (149 males and 75 females) in the lowest 25(OH)D quartile [serum 25(OH)D ≤ 24.38 nmol/L], based on a significance level of 0.05 for comparing the metabolic parameters.

### 2.4. Statistical Analysis

The data were analyzed with the Statistical Package for the Social Sciences (SPSS Inc., Chicago), Version 18.0. *P* < 0.05 was considered statistically significant. Normal distribution data was described as means ± SD. Considering that the distributions of FINS, HOMA-IR, and 25(OH)D were skewed, we used logarithmic transformations to normalize the distributions of these variables. A two-sample (independent group)* t*-test was performed to compare characteristics between the highest and lowest 25(OH)D quartiles. The relationship between 25(OH)D and insulin resistance was evaluated using Pearson correlation coefficient and multivariable linear regression analysis.

## 3. Results

### 3.1. Indexes Comparisons among 25(OH)D Categories

Of the enrolled subjects, with low serum 25(OH)D concentration, HOMA-IR, FINS, and SBP increased significantly (all *P* < 0.01).  Table 1 displayed that, compared with the lowest 25(OH)D quartile, individuals in the highest 25(OH)D quartile had Lg (HOMA-IR), Lg (FINS), and SBP decreased by 31.4%, 14.9%, and 4.4%, respectively (all *P* < 0.01) (Table 1).

### 3.2. Correlation and Regression Analyses of Vitamin D and Metabolic Indexes

Pearson correlation analyses (Table 2) showed that vitamin D was correlated with sex. The percentage of females in the lowest 25(OH)D quartile (75/149) was larger than the highest quartile (45/180). Serum 25(OH)D concentrations were negatively associated with age, BMI, Lg (HOMA-IR), and Lg (FINS), with coefficients −0.27, −0.11, −0.18, and −0.19, respectively, with all *P* < 0.01. No correlations were found between serum 25(OH)D and fasting plasma glucose (FPG), TG, TCH, HDLC, or LDLC. Multivariable linear regression analysis (Table 3) presented that sex, age, BMI, and Lg (HOMA-IR) were significant predictors of serum 25(OH)D concentrations (*P* = 0.001, 0.016, 0.002, and 0.023, resp.).

## 4. Discussion

Shaw et al. [15] once estimated that, between 2010 and 2030, there would be a 69% increase in the number of adults with diabetes in developing countries and a 20% increase in developed countries. This prediction suggests a larger burden of diabetes in the future, especially for undeveloped areas, including China with the largest population in the world. It has been gradually recognized that vitamin D could play a role in the pathogenesis of type 2 diabetes and its complications (such as diabetic nephropathy, diabetic retinopathy, and diabetic neuropathy) in recent years [16–18]. Insulin resistance is a recognized precursor in the development of type 2 diabetes. It will be useful to reduce the risk of type 2 diabetes by improving insulin resistance.

This study demonstrates a negative relation between serum 25(OH)D and insulin resistance in Chinese populations with NGT. This finding is in accordance with the previous reports [11, 19]. Liu et al. have demonstrated that, in Caucasian, higher vitamin D status was inversely associated with fasting glycemia and fasting markers of insulin resistance among individuals without diabetes [19]. The data from Chiu show a positive correlation of 25(OH)D concentrations with insulin sensitivity in 126 healthy, glucose-tolerant USA adults [11]. Besides, our findings are in agreement with another latest study conducted among healthy Chinese female people [8].

There are several suggested mechanisms for how vitamin D might influence insulin sensitivity. First, vitamin D insufficiency causes parathyroid hormone (PTH) to compensate for decreased calcium absorption, a response that leads to increased intracellular calcium, which may prevent insulin target cells from sensing the intracellular calcium fluxes necessary for insulin action [1]. Studies have shown that increased PTH was associated with impaired glucose tolerance and decreased insulin sensitivity [20]. Second, vitamin D may have an effect on insulin action through stimulating the expression of insulin receptors and enhancing insulin responsiveness for glucose transport [21]. Maestro et al. [22] demonstrated that treatment with 1,25(OH)_2_D_3_ for 24 hours increased in a dose-dependent manner the levels of the two major insulin receptor (IR) mRNAs (11 and 8.5 Kb) present in U-937 human promonocytic cells. Third, another study provided a link between VDR and PPAR signaling by identifying PPAR*δ* gene as a primary 1α, 25(OH)_2_D_3_ target gene. Meanwhile, PPAR*δ* had an effect on insulin sensitivity [23]. Finally, vitamin D may also be involved in inflammatory response and improve insulin sensitivity by directly modulating the effect of cytokines [24, 25]. Study on serum 25(OH)D and insulin resistance is insufficient in individuals with NGT, instead of prediabetes or participants with IFG [13, 14]. In this study, we observed that vitamin D independently negatively correlated with HOMA-IR after adjustment for possible confounders and those with low serum 25(OH)D levels were at high risk of multiple metabolic disorders. Just as revealed by Harinarayan et al. [26], adequate calcium-vitamin D supplementation might lead to improvement of insulin resistance and metabolism indexes in nondiabetic subjects with vitamin D deficiency. Recognizing the importance of vitamin D, vitamin supplement will be emphasized in clinical nursing work. Thus, defining a cutoff value of vitamin D deficiency based on insulin resistance would be significant [27]. Due to ethnic and dietary as well as age differences, different cutoff values may be detected in various regions.

Some limitations need to be noted. The cross-sectional study design used in this study cannot separate the timing of exposure and outcomes. Besides, a large-scale clinical experimental study and rigorous prospective study will be required.

In conclusion, serum 25(OH)D could be regarded as an independent predictor of insulin resistance for Chinese people with NGT. Further studies on 25(OH)D and insulin resistance will provide a new pathway for preventing the progression from normal glucose tolerance to type 2 diabetes.

## Figures and Tables

**Table 1:** Characteristics of the subjects by quartile categories of serum 25(OH)D.

	25(OH)D	
	The highest quartile	The lowest quartile
Cases	225	224
Sex (male/female)	180/45	149/75
BMI (Kg/m^2^)	24.47 ± 3.29	25.32 ± 2.78
Age (y)	45.72 ± 7.10	51.75 ± 6.12
FPG (mmol/L)	5.58 ± 0.57	5.59 ± 0.49
Lg (HOMA-IR)	0.42 ± 0.29	0.61 ± 0.31**
LgFINS (mIU/L)	1.03 ± 0.28	1.21 ± 0.30**
SBP (mmHg)	128.72 ± 1.53	134.70 ± 1.61**
DBP (mmHg)	84.96 ± 0.95	81.69 ± 1.11
TG (mmol/L)	1.76 ± 0.09	2.01 ± 0.14
TCH (mmol/L)	5.40 ± 0.08	5.64 ± 0.09
HDLC(mmol/L)	1.22 ± 0.27	1.24 ± 0.28
LDLC (mmol/L)	3.34 ± 0.82	3.35 ± 0.85

∗∗ indicates *P* < 0.01, compared with the highest quartile.

**Table 2:** The correlation and regression analyses of Lg (vitamin D).

	Pearson correlation		Stepwise regression	
	*r*	*P*	*β*	*P*
Sex	0.20	0.000	0.140	0.001
Age	−0.27	0.000	−0.248	0.000
BMI	−0.11	0.008	−0.130	0.002
SBP	−0.11	0.007	−0.010	0.820
DBP	−0.10	0.018	−0.009	0.845
Lg (HOMA-IR)	−0.18	0.000	−0.183	0.000
lgFINS	−0.19	0.000	0.128	0.657

**Table 3:** The variables entering into the multivariate linear regression equation.

Variables	Partial regression coefficient (*B*)	Standardized partial regression coefficient (*β*)	Standard error	*T* value	*P* value
Constant	1.166		0.071	16.362	0.000
Sex	0.054	0.140	0.001	3.441	0.001
Age	−0.006	−0.248	0.016	−6.252	0.000
BMI	0.007	−0.130	0.002	−3.059	0.002
Lg (HOMA-IR)	−0.104	−0.183	0.023	−4.505	0.000

Lg 25(OH)D as dependent variable.
